# Invasive adenocarcinoma arising from a mixed hyperplastic/adenomatous polyp and synchronous transverse colon cancer

**DOI:** 10.1186/1477-7819-11-214

**Published:** 2013-08-28

**Authors:** Chuang-Wei Chen, Koung-Hong Hsiao, Chung-Tai Yue, Chia-Chi Wang

**Affiliations:** 1 Division of Colon and Rectal Surgery, Department of Surgery, Taipei Tzu Chi Hospital, Buddhist Tzu Chi Medical Foundation, Taipei, Taiwan; 2 School of Medicine, Tzu Chi University, Hualien, Taiwan; 3 Department of Pathology, Taipei Tzu Chi Hospital, Buddhist Tzu Chi Medical Foundation, Taipei, Taiwan; 4 Department of Gastroenterology, Taipei Tzu Chi Hospital, Buddhist Tzu Chi Medical Foundation, 289 Road, Taipei, Xindian City 23142, Taiwan

**Keywords:** Adenocarcinoma, Colon, Hyperplastic, Adenomatous, Polyp

## Abstract

An admixture of hyperplastic and adenomatous components within the same polyp is unusual. Adenocarcinoma arising from a mixed hyperplastic/adenomatous polyp (MHAP) occurs even more rarely. We report the first case of a 59-year-old male who presented with invasive adenocarcinoma originating from a MHAP at a sigmoid colon and synchronous transverse colon cancer.

## Background

As we know, hyperplastic polyps are rarely precursors of malignancy and play no role in adenoma-carcinoma sequence [1]. However, coexistence of hyperplastic and adenomatous components within a polyp has been documented in the literature [2]. To our knowledge, only two cases of adenocarcinoma originating from a mixed hyperplastic/adenomatous polyp (MHAP) have been previously reported. Herein, we report a case of invasive adenocarcinoma arising from a MHAP at a sigmoid colon and synchronous transverse colon cancer.

## Case presentation

**Figure 1:**
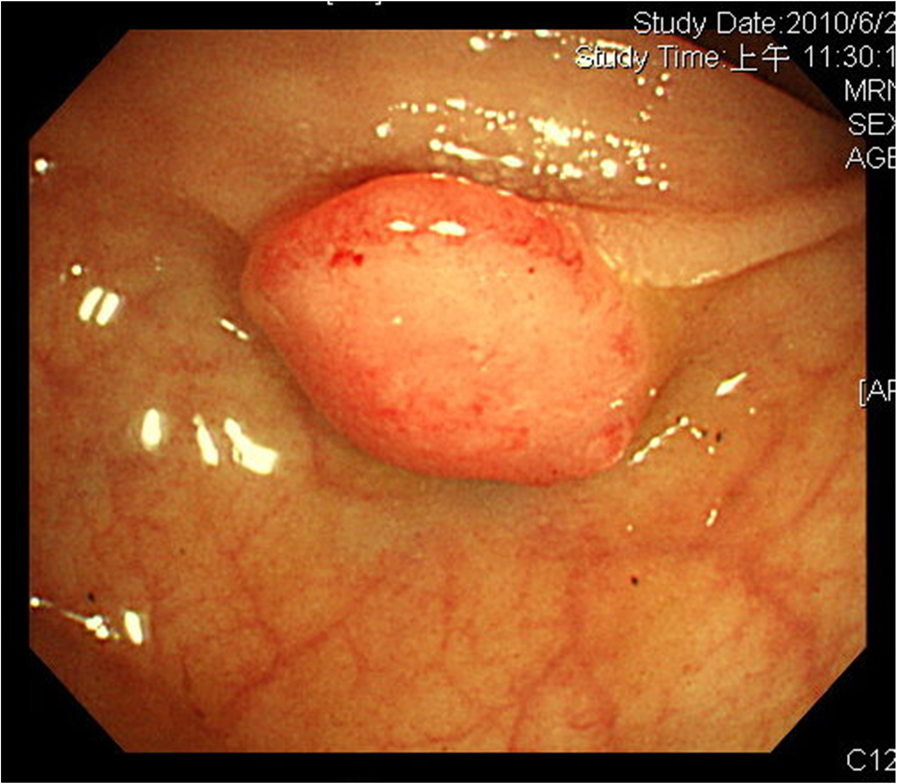
Colonoscopy showed a 1 cm sessile polyp of the sigmoid colon.

**Figure 2:**
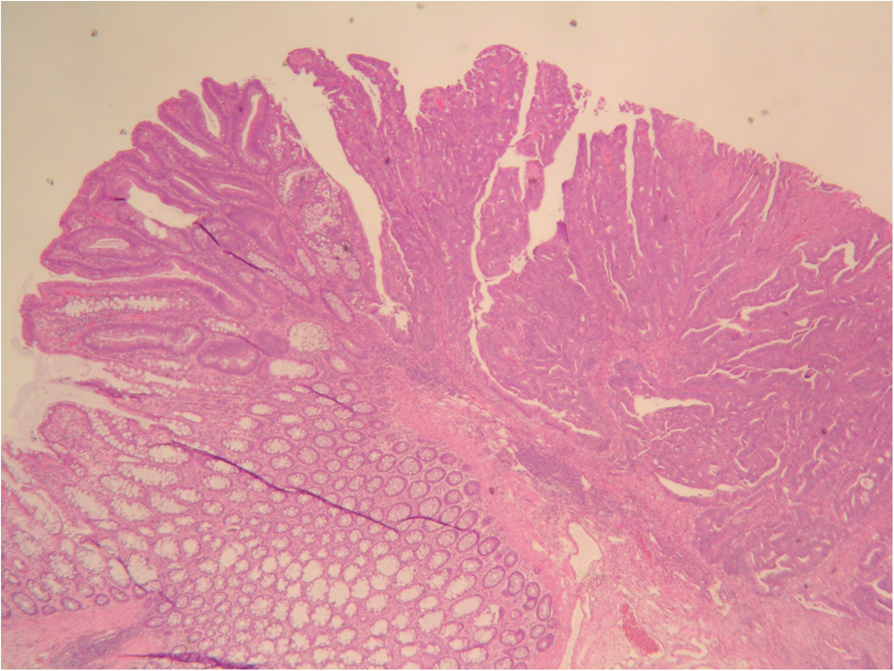
**Transition from hyperplastic (left lower) to adenomatous (left upper) to carcinomatous (right) regions.** (H & E stain × 20).

A 59-year-old Chinese man had superficial bladder cancer treated by transurethral resection and intravesical biological therapy half a year previously. He presented with a one-month history of abdominal cramping pain and anemia. There was no family history of colon or breast cancer. Laboratory examinations disclosed his hemoglobin was 9 g/dL and his carcinoembryonic antigen (CEA) was 2.16 ng/ml (normal: 0 to 5). A computed tomography (CT) scan found wall thickening of the proximal transverse colon and diverticulosis at the ascending colon. Subsequent colonoscopy showed an annular tumor at the proximal transverse colon and a 1 cm sessile polyp at the sigmoid colon (Figure 1). Biopsy was done at the transverse colon tumor and histology confirmed adenocarcinoma. Snare polypectomy for the sigmoid colon polyp was performed and histology revealed an invasive adenocarcinoma arising from a mixed hyperplastic/adenomatous polyp (MHAP) (Figure 2). The final diagnosis was synchronous transverse colon cancer and invasive adenocarcinoma arising from a mixed polyp of the sigmoid colon. Because the resected sigmoid polyp showed stromal invasion, lymphatic invasion and a free margin of less than 1 mm in histology, right hemicolectomy and sigmoidectomy were undertaken simultaneously. Surgical pathology revealed a T3N0 adenocarcinoma of the proximal transverse colon and no residual cancer was found at the sigmoid colon. The patient had an uneventful recovery and received adjuvant chemotherapy. No recurrence was noted during 12 months of follow-up.

## Conclusions

It is generally accepted that hyperplastic polyps are not precursors of malignancy and play no role in adenoma-carcinoma sequence [1]. However, an admixture of hyperplastic and adenomatous components within a polyp has been documented [2]. Besides the two main pathways of adenoma-carcinoma sequence and DNA microsatellite instability (MSI) related to development of colorectal cancer, emerging evidence suggested a third serrated neoplastic pathway [3,4]. The morphological spectrum of polyps with serrated architecture includes traditional hyperplastic polyps, mixed hyperplastic adenomatous polyps and serrated adenoma. Recent studies indicate that mixed hyperplastic polyps contain a coexistence of serrated adenoma and hyperplastic components within the same polyp and are likely to be heterogeneous at a molecular level with an independent histogenetic pathway that may be underestimated as a premalignant lesion [4,5].

To the best of our knowledge, only two cases of adenocarcinoma originating from a MHAP were reported in the literature. Despite being uncommon, it may be an entity that is underestimated. Herein, we report a case of invasive adenocarcinoma arising from a MHAP at a sigmoid colon and synchronous transverse colon cancer. Some things learned from this case should be emphasized. First, it is difficult to make a clear distinction among pure hyperplastic polyps, adenomatous polyps or mixed polyps by endoscopic features. Thus, endoscopic polypectomy with a free margin is suggested whenever possible. Second, careful pathologic examination of the resected polyps is recommended since the adenomatous component with dysplasia, even the infiltrating adenocarcinoma, may coexist within the hyperplastic polyp. Third, this case with synchronous lesions provides evidence that patients with MHAP have a risk of developing advanced colon cancer.

## Consent

Written informed consent was obtained from the patient for the publication of this report and any accompanying images.

## Abbreviations

CEA: Carcinoembryonic antigen; CT: Computed tomography; MHAP: Mixed hyperplastic/adenomatous polyp; MSI: Microsatellite instability.

## Competing interests

The authors declare that they have no competing interests.

## Authors’ contributions

CCW: writing the manuscript. HKH: provide opinion. YCT: pathologic figure. WCC: revise paper. All authors read and approve the final manuscript.
